# Loss of SMYD1 Results in Perinatal Lethality via Selective Defects within Myotonic Muscle Descendants

**DOI:** 10.3390/diseases7010001

**Published:** 2018-12-20

**Authors:** Tara L. Rasmussen, Haley O. Tucker

**Affiliations:** 1 Department of Molecular Biosciences, the University of Texas at Austin, Austin, TX 78712, USA; tara.rasmussen@bcm.edu; 2 Department of Molecular Physiology and Biophysics, Baylor College of Medicine, Houston, TX 77030, USA

**Keywords:** cardiovascular biology, histone methyl transferase, myogenic regulation, fate determination

## Abstract

SET and MYND Domain 1 (SMYD1) is a cardiac and skeletal muscle-specific, histone methyl transferase that is critical for both embryonic and adult heart development and function in both mice and men. We report here that skeletal muscle-specific, *myogenin (myoG)-Cre*-mediated conditional knockout (CKO) of *Smyd1* results in perinatal death. As early as embryonic day 12.5, *Smyd1* CKOs exhibit multiple skeletal muscle defects in proliferation, morphology, and gene expression. However, all myotonic descendants are not afflicted equally. Trunk muscles are virtually ablated with excessive accumulation of brown adipose tissue (BAT), forelimb muscles are disorganized and improperly differentiated, but other muscles, such as the masseter, are normal. While expression of major myogenic regulators went unscathed, adaptive and innate immune transcription factors critical for BAT development/physiology were downregulated. Whereas classical mitochondrial BAT accumulation went unscathed following loss of SMYD1, key transcription factors, including PRDM16, UCP-1, and CIDE-a that control skeletal muscle vs. adipose fate, were downregulated. Finally, in rare adults that survive perinatal lethality, SMYD1 controls specification of some, but not all, skeletal muscle fiber-types.

## 1. Introduction

Skeletal myogenesis is a critical biological process that is essential for development, regeneration, and homeostasis [1,2]. A number of key transcription factors (TFs) control skeletal muscle development, including regulators of progenitors (e.g., PAX3 and PAX7), regulators of myogenic commitment (e.g., MYF5, MYOD1, and MYF6), and regulators of embryonic myocyte fusion (e.g., MYOG). These and other TFs act as a complex and often compensatory overlapping network to regulate both spatial and temporal expression. 

SET and MYND Domain 1 (SMYD1) is a cardiac and skeletal muscle-specific, chromatin-binding factor that regulates growth and morphogenesis during fetal and neonatal development in mice [3,4], humans [5,6], and zebrafish [7,8]. SMYD1 is expressed within the heart and within fast-twitch skeletal muscle cells [3,4,5,6]. SMYD1 family members catalyze methylation of histone H3 lysine K4 in vitro, and as initially suggested by Gottlieb et al. [3], act within the developing heart as transcriptional repressors in vivo [4,5,6]. In some cases [4], SMYD1 inhibits the transcription of target genes by binding directly to target promoters. Alternatively, SMYD1 can serve as a direct target of TFs, including serum response factor (SRF) and MYOG, to direct sarcomere function in vivo [9]. Within sarcomeres, SMYD1 localizes to the M-line, where it physically associates with myosin [10], and within the nucleus, where it mediates transcriptional repression. SMYD1 also is expressed in the cytoplasm, where it has been implicated in aspects of adult heart development and oxidative stress [4,11]. 

SMYD1 is expressed in somitic myoblasts as early as E9.5, and its expression persists throughout myogenesis and into mature myofibers [3,12]. While SMYD1 function in the early developing heart has been well documented [3,5,11], its function in early skeletal myogenesis is less understood. By employing *Myf6-Cre*, we ablated *Smyd1* following myofiber differentiation in skeletal myocytes [12,13]. Mutant mice appeared normal through gestation, but when examined ~6 weeks postnatally, they exhibited a phenotype similar to non-degenerative myopathy. Defects included physical weakness, myofiber hypotrophy, and a prevalence of oxidative myofibers. While conditional knockout (CKO) mice showed no evidence of re- or degenerating myofibers, they upregulated muscle developmental genes and lost fast-twitch muscle markers. 

Since the onset of *Myf6-Cre*-induced recombination initiates in skeletal muscle at ~E17.5 and maximizes near birth [14], we sought to address the impact of *Smyd1* disruption within the interval between E9.5 and term. *Myogenin-Cre* (*MyoG-Cre*) expression can be detected modestly within inter-limb somites at E9.75–E10.75 [15]. From E11.75 onward, *Myog-Cre* directs increased excision within the trunk as well as proximal and distal limb muscles. By E15.25, deletion covers the dorsal trunk surface and most subcutaneous muscles, albeit mosaic expression of *MyoG-Cre* in hind limb muscle groups has been documented [16]. 

We report here that skeletal muscle-specific, *MyoG-Cre*-mediated conditional knockout (CKO) of *Smyd1* results almost exclusively in perinatal death, which is unexpected, given only a single previous report of *Myog-Cre*-mediated lethality [17]. *Smyd1* CKOs exhibit multiple skeletal muscle pathologies, but not all myotonic descendants are afflicted equally. While expression of Muscle Regulatory Factors (MRFs) and other previously observed transcriptional regulators were unaffected, TFs controlling adaptive and innate immune processes underlying BAT development/physiology as well as PRDM16, a TF that determines muscle vs. adipose fate, were deregulated. In rare *Smyd1* CKO adults that survived perinatal lethality, muscle fiber type specification was perturbed. These findings further expand the breadth and scope of SMYD1 methyltransferase function in skeletal muscle biology. 

## 2. Methods and Materials

### 2.1. Generation of Myog-Cre/Smyd1^flox^ Mice

All protocols involving animals conformed to the NIH Guide for the Care and Use of Laboratory Animals and were approved by the University of Texas Animal Research Committee (Institutional Animal Care and Use Committee, #AUP-2010-00097; NIH Assurance #A4107-01). *Smyd1flox* mice were generated as detailed by Gottlieb et al. [3], as summarized below.

### 2.2. Mating Scheme

*MyoG-Cre* mice were provided by Dr. Eric Olson as F1 first generation hybrids of C3H and C57BL/6J. Recombination induced by *MyoG-Cre* is initiated in anterior somites at E9.0, and by E9.5, expression is detected in all somites and is confined and maintained in muscle tissue throughout embryogenesis and into adulthood [18]. The same breeding scheme as described previously [3] was employed to create *Smyd1^Flox/KO^*; *MyoG-Cre*^+/−^ mice (BL6.FVB.129.C3H hybrids) as well as the following breeders of the same background: *Smyd1^Flox/WT^*; *MyoG-Cre*^+/−^ and *Smyd1^KO/WT^*; *MyoG-Cre*^+/−^. However, in order to avoid breeding *Cre* to homozygosity, we began crossing the above genotypes with *Cre*-negative mice harboring the *Smyd1^Flox^* and *Smyd1^KO^* alleles. It later became apparent that *Smyd1^Flox/Flox^*; *MyoG-Cre*^+/−^ and *Smyd1^Flox/KO^*; *MyoG-Cre*^+/−^ displayed the same phenotype. In order to simplify the number of genotypes created and to create higher numbers of interesting genotypes, we mated Smyd1^Flox/Flox^ to *Smyd1^Flox/WT^*; *MyoG-Cre*^+/−^. These mice were only crossed to one another, thereby maintaining an inbred, but non-isogenic, hybrid population that was similar, but isolated, from the other *Cre*-induced CKO lines.

### 2.3. PCR Genotyping

At weaning, each mouse was given a unique ear punch and a short piece of tail (2–5 mm) was biopsied. The tail tissue was cooked overnight at 55–60 °C in 200 µL tail PCR buffer (50 mM KCl, 10 mM Tris (pH 8.3), 2.5 mM MgCl_2_, 0.1 mg/mL gelatin, 0.45% *v*/*v* NP40, 0.45% *v*/*v* Tween 20) plus 2 µL proteinase K (10 mg/mL), boiled for 5 min, and centrifuged for 10 min. PCR conditions were as follows: Step 1—94 °C for 5 min; Step 2—94 °C for 30 s; Step 3—62 °C for 1 min; Step 4—72 °C for 1 min; Step 5—repeat Step 2 29 times; Step 6—72 °C for 7 min; Step 7—4 °C for storage. The primers for detection of *Smyd1^WT^* and *Smyd1^Flox^* were: 5′-cacatctttggtgtggtatggc-3′ and 5′-ctcacttgcgtcccagtacttg-3′ (amplified product, 540 bp and 620 bp); 5′-gcatacgcacatgtgctcgc-3′ and 5′-tcatgagatgggcatgagcc-3′ (432 and 552 bp); *Smyd1^KO^* Genotyping primers: 5’-tcatgagatgggcatgagcc-3′ and 5′-gcccggttctttttgtcaagaccga-3’ (985bp). Genotyping primers for Cre were: 5′-ggacatgttcagggatcgccaggcg-3′ and 5′-gcataaccagtgaaacagcattgctg-3′ (268 bp). 

### 2.4. Recombination Assays

Recombination in each cross was determined by PCR genotyping to determine tissue specificity and efficiency of deletion. Heterozygous mice (*Smyd1^Flox/WT^*; Cre^+/−^) were sacrificed and tissues were harvested and then incubated at 55–60 °C in PCR buffer (50 mM Tris pH 8.0, 100 mM EDTA, 100 mM NaCl, 1% SDS) and 70 µL proteinase K (10 mg/mL). After complete digestion (16–48 h), samples were phenol/chloroform extracted, aqueous phase extracted and then centrifuged for 15 min. Pellets were washed in 70% ethanol, then dried and resuspended in water at 37 °C. Aliquots used for genomic PCR were subjected to the following program: Step 1—94 °C for 5 min; Step 2—94 °C for 30 s; Step 3—62 °C for 1 min; Step 4—72 °C 1 min; Step 5—return to step 2 for 20–30 cycles using primers: 5′-cacatctttggtgtggtatggc-3′ and 5′-ctcacttgcgtcccagtacttg-3′ to detect *Smyd1^WT^* (540 bp) and *Smyd1^Flox^* (620 bp), respectively; 5′-tcatgagatgggcatgagcc-3′ and 5′-gcatacgcacatgtgctcgc-3′ to detect *Smyd1^WT^* (432 bp) and *Smyd1^Flox^* (552 bp), respectively. *Smyd1^Floxdel^* produced a band of 395 bp.

### 2.5. In Situ Hybridization

Embryos at 15.5 days post coitum (dpc) or younger were extracted in DEPC PBS (1 mL DEPC/L, autoclaved) and tail pieces were removed for genotyping. The remainder of the embryo was fixed in at 10-fold volumes of 4% paraformaldehyde at 4 °C on a rotator overnight. Samples were then washed and either stored at 4 °C or embedded in paraffin for tissue sectioning as previously described [19].

### 2.6. Protein/Immunohistochemistry

Embryos and adult tissues were extracted in PBS and fixed in at least 10-fold volumes of 10% formalin or 4% paraformaldehyde at room temperature on a rotator for 24–72 h. Embryos older than 15.5 dpc were skinned before fixation. Tail segments prior to fixation were genotyped. Samples were washed three times for at least 1 h in PBS and either stored at 4 °C or prepared for paraffin embedding and tissue sectioning as previously described [19].

### 2.7. End-Point RT-PCR

Trizol homogenates were thawed and RNA was isolated according to the Invitrogen Trizol protocol. For embryonic samples, isopropanol precipitation was supplemented with 5–10 µg of RNAse free glycogen. After drying the RNA pellet, samples were resuspended in 25 µL deionized DEPC water and immediately DNase1 treated with 15 µL mix D (2.7× DNase I buffer (Invitrogen, Carlsbad, CA, USA); 1 U/µL RNaseOut (Invitrogen, Carlsbad, CA, USA); 2 U/µL DNase I (Roche, Indianapolis, IN, USA)) to a final volume of 40 µL and incubated at 37 °C for 1 h. The enzyme was heat inactivated in 4 µL of 25 mM EDTA and incubation at 70 °C for 10 min. RNA quality and concentration were analyzed using a nanodrop spectrophotometer. Samples were boiled for 5 min and iced for 5 min before adding 13 µL of mix 2 (3.8× 1st Strand Buffer, 20 mM DTT, 1 U/µL RNaseOut (Invitrogen, Carlsbad, CA, USA)). After pre-warming the reaction mixture for 2 min at 37 °C, 2 µL of 200 U/µL MMLV (Invitrogen, Carlsbad, CA, USA) was added to each reaction except the negative control. Samples were reverse transcribed for 1.5 h at 37 °C. The enzyme was heat inactivated at 85 °C for 5 min and the samples were stored at 4 °C. Next, 1–2 µL of the cDNA reaction were used for RT-PCR with various gene primer sets (Figure S1) or with primers for *Smyd1*: 5′-ctgcgggcagtgcaagtt-3′ and 5′-gctcacaggagcagtcaaagtag-3′; β-actin: 5′-tacgagggctatgctctc-3′ and 5′-cgcagctcagtaacagtc-3′. 

### 2.8. RT-qPCR 

Total cellular RNA was isolated using an RNeasy Mini Kit (QIAGEN, Santa Clara, CA, USA). cDNA synthesis was performed with qScript cDNA supermix (Quanta Biosciences, Inc., Denver, CO, USA). RT-qPCRs were performed using PerfeCTa SYBR Green FastMix (Quanta) with 1 µL of 20×-diluted cDNA generated from 500 ng of total RNA. RT-qPCR primers were designed to amplify the junction between two exons. Primer sequences for qPCR are listed in Figure S2. Ct values were normalized against GAPDH. qPCR was performed with an initial denaturation step of 10 min at 95 °C, followed by 15 s at 95 °C and 60 s at 60 °C for 30 cycles using an MX3000P™ QPCR system (Stratagene, La Jolla, CA, USA). The normalized level of a mRNA was determined as 2−Ct(GOI)/2−Ct(CTL), where Ct is the threshold cycle, GOI is the transcript of interest, and CTL is the housekeeping control (assuming that Ct is inversely proportional to the initial concentration of mRNA and that the amount of product doubles with every cycle). The statistical significance of RT-qPCR results was determined by two-tailed *t*-test in Microsoft Excel. All sample values were normalized to GAPDH levels (primer sequences noted in Figure S2). GAPDH levels were not significantly altered among different groups (data not shown).

### 2.9. RNA In Situ Hybridization 

Embryos were harvested at various pre- and postnatal gestation times, fixed in 4% paraformaldehyde overnight, and in situ hybridization was performed by whole-mount hybridization as described previously [3,19]. Following pre-hybridization procedures, tissue sections were hybridized at 55 °C with a sense and antisense riboprobe for murine Myogenin. Briefly, RNA antisense *MyoD* was obtained by RT-PCR after denaturing RNA for 10 min at 70 °C in PCR buffer (2.5 mM MgCl2), 1 mM dNTPs, 50 ng of oligo primer (dT) (Gibco, Carlsbad, CA, USA) and 200 units of the reverse transcriptase enzyme Superscript II (Gibco, Carlsbad, CA, USA). Primers used for the amplifications designed from a combination of the complementary *MyoD* gene sequence with the T7 bacteriophage promoter sequence in its 5′ end were 5′-agcactacagtggcgactca-3′ and 5′-ggccgctgtaatccatca-3′. Hybridization was conducted overnight with antisense and sense probes (Figure S2) using ~7.5 × 10^5^ disintegrations per minute(dpm) per slide. The unhybridized probe was removed through stringent washes and treatment with RNase A. Slides were subsequently coated with K.5 nuclear emulsion (Ilford, UK) and exposed at 4 °C for 12 days. Slides were developed, counter-stained with hematoxylin, and examined using bright and dark field optics.

## 3. Results and Discussion

### 3.1. MyoG-Cre Deletion is Specific to Skeletal Muscle

**Figure 1:**
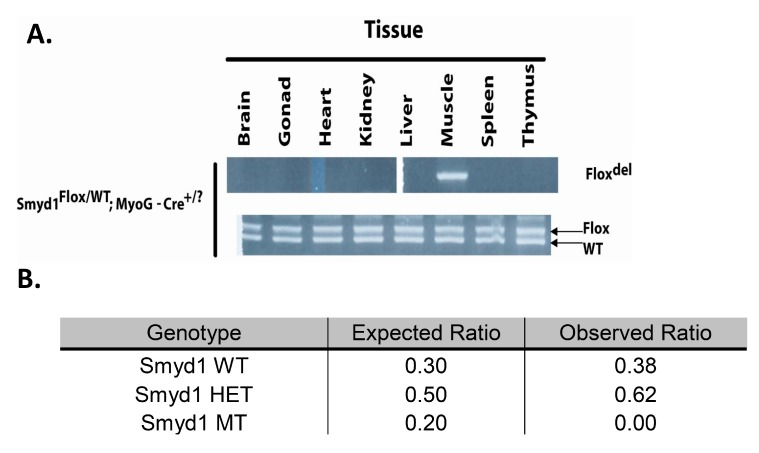
*MyoG-Cre* deletion of *Smyd1* is muscle-specific lethal. (**A**) *MyoG-Cre* induces recombination of *Smyd1* specifically in muscle. DNA was prepared from the tissues of a heterozygous *Smyd1* mice. Recombination was assayed in the upper panel and the positive control *Flox/WT* assay was assayed in the lower panel. (**B**) Skeletal muscle-specific *Myog-Cre/Smyd1* conditional knockout (CKO) is lethal prior to weaning. Various Smyd1^Flox/KO/WT^/MyoG-Cre crosses obtained an N of 350. We expect that 20% of pups to be homozygous for *Smyd1*-deficient alleles, 50% of pups should be heterozygous, and 30% should be wild type (*WT*). Litters were genotyped at weaning and the observed numbers were recorded. There was no significant reduction in litter size between birth and weaning.

As described in the Materials and Methods section, we analyzed *Cre* excision fidelity for genomic DNA recombination of the *Smyd1* locus in heterozygous mice. As shown in Figure 1A, there was no detectable deletion of the *Flox* compared to the wildtype (*WT*) allele, but efficient recombination was observed specifically within skeletal muscle tissue. 

### 3.2. MyoG-Cre-Mediated Smyd1 CKO Results in Perinatal Lethality 

Unexpectedly, of the first 350 pups weaned and genotyped at 3–6 weeks, there were zero *Smyd1* conditional knockouts (CKOs; Figure 1B). Dead pups were found during and shortly after birth, but significant numbers of pups did not disappear between postnatal day 1 and weaning. The birth of a few litters was observed. CKO pups were difficult births, probably because of their increased size due to extreme edema. These pups were often crushed during their birthing process. Those that were born intact showed no signs of movement nor breathing. 

Genotypic ratios for these litters were analyzed from E9.5 to E17.5, and no significant variance from the expected Mendelian ratios were observed (Table 1). Fetuses were extracted at E18.5—an age at which they could be fostered and survive if viable. However, unlike their littermates, *MyoG-Cre*-induced CKOs at this age made no attempts at locomotion nor breathing. Thus, CKOs were not dying simply due to birthing problems, but never showed signs of life during late stage embryogenesis. Whether or not the heart was pumping at this age was not determined, but without locomotion, these embryos could not survive. 

Therefore, the CKOs are perinatal lethal—a result unanticipated given that only a single previous example of *MyoG-Cre*-induced lethality had been observed [17]. We return to this point in the present context below. 

### 3.3. Skeletal Muscle Development in the MyoG-Cre-Induced Smyd1 CKO is Significantly Derailed 

Viable embryos initially were inspected visually from E11.5 through E18.5 for gross defects. At E12.5, embryos appear normal after sectioning (data not shown). Some embryos were outwardly abnormal as early as E13.5, but by E15.5, CKOs were easily discerned from littermates by eye. 

**Figure 2:**
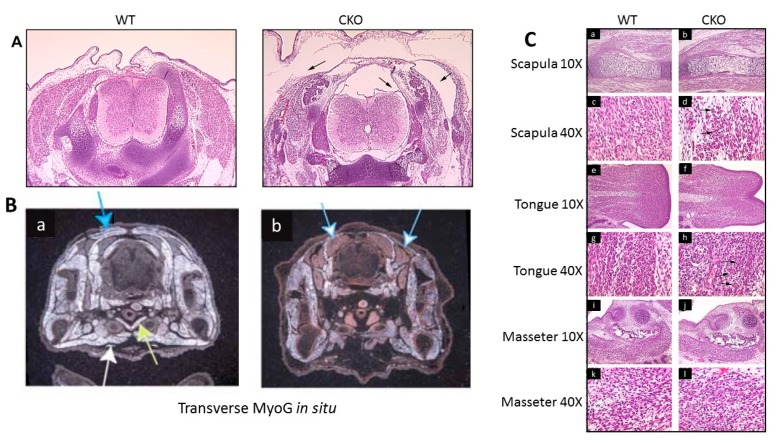
Skeletal muscle development in the *MyoG-Cre*-induced *Smyd1* CKO is significantly derailed. (**A**) Haematoxylin and Eosin (H&E)-stained transverse sections of E14.5 WT and CKO embryos stained with H&E. Marked subcutaneous edema is most visible in the cervical region (arrows). (**B**) Myogenin in situ hybridization of WT and CKO transverse sections reveal reduction of myocytes and general affliction of some, but not all, muscles. The pectoral (white arrow) and sternohyoid (yellow arrow) muscles did not develop in the CKO; the dorsal muscles (solid blue arrow) are significantly diminished, but other muscles (e.g., limbs) are clearly less afflicted, if at all. There is an excess of tissue tentatively classified as brown adipose tissue (blue and white arrows) in the CKO. (**C**) H&E stains (**a**–**h**) of WT and CKO sections at E15.5. Scapulae (**a**–**d**) show asynchronous elongation and emergence of large, multinucleated cells—myoblasts that did not fuse—but failed to elongate into myotubes (black arrows in **d**). Stains of CKO tongues (**e**–**h**) displayed the same phenotypes as above with evident multinucleated, unfused structures (black errors in **h**). CKO masseter muscles (**i**–**l**) had no observable morphologic defects.

In order to analyze potential skeletal muscle disruption in detail, CKO pups at various embryonic ages were sectioned and compared to their WT littermates. At E14.5, Haematoxylin and Eosin (H&E)-stained transverse sections of CKOs displayed a marked subcutaneous edema most visible in the cervical region (arrows in Figure 2A and data not shown). 

Next, we performed in situ hybridization on transverse sections employing as probe, Myog, which marks all myogenic lineages, on sections prepared at E14.5 (Figure 2B). While the sections are not matched precisely in the rostro-caudal plane, there is a clear reduction of Myog-marked myofiber density in the CKO. However, muscle groups were affected differently. For example, at E15.5, CKO limbs displayed less cellularity than controls, in correspondence with the Myog in situ hybridization (Figure 2B). Unanticipatedly, the pectoralis (white arrow) and sternohyoid muscles (yellow arrow) were no longer visible in sections; i.e., missing entirely (Figure 2B). We also noted that the dorsal muscles (solid blue arrows) were significantly diminished, while other muscles, including the limbs, were clearly less affected. There also appeared to be an excess of brown adipose tissue (blue and white arrows) in the CKO (Figure 2B and re-addressed below).

Failure of CKO muscular development also can be seen in the H&E-stained cross-sections of Figure 2C. For example, the scapula region revealed that the surrounding muscle cells were less dense and less organized (Figure 2(Ca–d)). Also, there was an emergence of quite large, multinucleated cells—myoblasts that had fused, but apparently had failed to elongate into myotubes (marked with arrows in Figure 2(Cd)). CKO tongues displayed the same phenotypes as scapula muscles, with evident multinucleated, unfused structures, particularly evident in the CKO tongue (Figure 2(Ch), arrows). However, the masseter muscles appeared totally unscathed from observable morphologic defects (Figure 2(Ci–l)). 

In summary, SMYD1 skeletal muscle deficiency does not affect all myotonic descendants equally. Trunk muscles are nearly ablated, forelimb muscles are disorganized and improperly differentiated, but some muscles, such as the masseter, are normal. We observed that muscles appear normal as late as E12.5, following post-migration from the somitic myotome (data not shown). However, it is possible that the molecular effect occurred earlier than the visible effect, and *Smyd1* is involved in some essential event(s) prior to this migration. Finally, TUNEL staining on sections of WTs and CKOs detected no difference in the number of apoptotic cells (data not shown). 

These results along with the increased numbers of large multinucleated cells near areas of reduced cell density suggest that the paucity of myofibers is secondary to degeneration. However, we cannot distinguish whether the myofibers did not form or if they instead underwent rapid degeneration.

### 3.4. MyoG-Cre-Induced Smyd1 CKOs Exhibit Proliferative Defects and Non-Autonomous Deposits of Brown Adipose Tissue 

**Figure 3:**
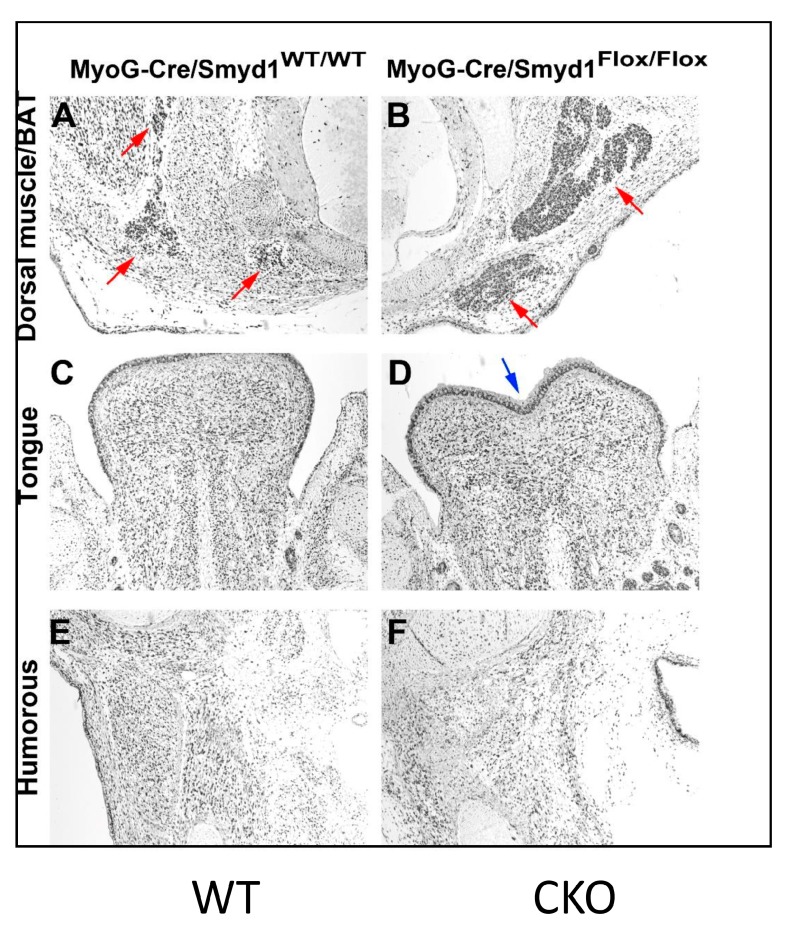
*MyoG-Cre*-induced *Smyd1* CKOs have proliferative defects and non-autonomous deposits of brown adipose tissue. (**A**,**B**). Reduced proliferation within E14.5 embryonic tissue and accumulation of brown adipose tissue (BAT, pink arrows) within dorsal muscles as detected by reduced Ki67 staining in CKO. (**C**,**D**) Proliferation differences within the epidermal region at the tip of the CKO tongue. The line of proliferation lags in the CKO (**D**, blue arrow) as compared to WT (**C**). (**E**,**F**) Insignificant proliferative differences within humerus muscles of WT and CKOs.

To determine whether the apparent reduction in cellular density is due to a decrease in proliferation, sections were analyzed by immunohistochemistry for Ki67, a protein present in all active, but not in quiescent, stages of the cell cycle [20]. CKO skeletal muscles analyzed at E12.5 showed normal proliferation (data not shown). However, at E14.5, we observed reduction in Ki67 staining in dorsal sections (Figure 3A,B) but not in muscles surrounding the CKO humorous (Figure 3E,F). Notably, the leading edge of proliferation within the CKO tongue epithelium is significantly lagging and appears to be anatomically misshaped (blue arrow in Figure 3D).

We suspected that the Ki67 dense regions (particularly evident in CKO dorsal muscle (red arrows)) were fat deposits. Adipocytes can be divided into classes of white (WAT), brown (BAT), or beige cells [21]. We tentatively assigned these adipocytes as “standard” BAT based on: (1) the eosin component of H&E stain distinguishes WAT from BAT (i.e., eosin colors BAT, but not WAT); (2) unfixed CKO cryostat sections were enriched in deposits staining positively with Sudan Black—a dye specific for BAT (data not shown). 

In CKOs, BAT deposits were both increased as well as more heavily stained with H&E (pink arrows in Figure 3A,B). The CKO BAT area appears to be surrounded by other smaller cells—most likely lymphocytes and other immune cells. BAT composition, function, and numbers undergo significant changes in response to nutritional and mitochondrial demands (readdressed below).

**Figure 4:**
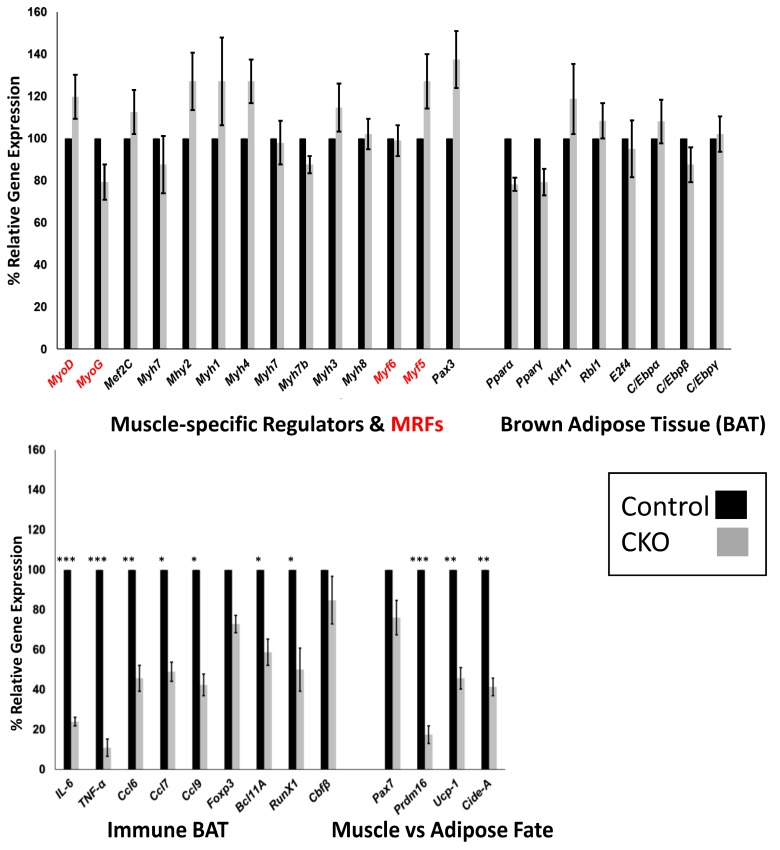
E12.5 *Smyd1* skeletal muscle loss results in transcriptional dysregulation of genes required for development of immunologic brown adipose tissue and genes regulating adipose vs. skeletal muscle fate. Transcription of muscle specific regulators (black) including Muscle Regulatory Factors (MRFs) (red) or regulators of conventional brown adipose tissue (BAT) are not altered in *Smyd1/MyoD-Cre* (CKO) E12.5 embryos. Factors critical to BAT formation within the immune system (Immune BAT) and factors critical to progenitor differentiation to skeletal muscle or adipose tissue (Muscle vs. Adipose Fate) are significantly altered in the CKO relative to WT. E12.5 embryo preparation, RNA preparation, RT-qPCR, and associated analyses were performed as detailed in the Materials and Methods section employing primer-pairs listed in Figure S2. Data from three biological replicates were averaged. Absolute quantification of each target was performed using a standard curve as a reference (Roche Light Cycler software version 1.5, Pleasanton, CA, USA). PCR primers were designed using Primer3 software [23,24]. Significance was determined by student’s *t*-test as: * *p* ≤ 0.05; ** *p* ≤ 0.01; *** *p* ≤ 0.001.

Excess BAT was confirmed in other tissue sections (e.g., Figure 2B,C). Perhaps relevant in this context was our observation that, following shRNA knockdown of SMYD1 in C2C12 myocytes, higher levels of triglycerides—a component of adipose tissue—were apparent (L. Zhu and H.O. Tucker, data not shown). However, a previously considered essential BAT regulator, Prdm16, was downregulated—the opposite effect of that which was predicted (Figure 4).

### 3.5. Skeletal Muscle SMYD1 Deficiency Does not Affect Transcript Levels of Myogenic Regulatory Factors

In an attempt to identify pathways disrupted by skeletal muscle loss of *Smyd1*, we analyzed the transcript pool at E12.5 in an attempt to preclude secondary and indirect effects. Because the myogenic program was significantly derailed, we first examined 14 muscle-specific TFs, including the helix-loop-helix regulatory factors (MRFs) MYOG, MYOD1, MYF5, and MYF6 [22]. As shown both by real time RT-qPCR (Figure 4) and by endpoint RT-PCR at E13.5 (Figure S3), no significant changes in expression were observed.

Given the severity of the phenotype, this result was unanticipated. However, MRFs also were not targeted in the only other reported case of *Myog-Cre*-induced lethality [17]. Instead, the target of *Myog-Cre* was the *Mef2c* muscle enhancer, whose lethality occurred perinatally or postnatally (P) depending on genetics [17]. In a C57BL/6 mixed background, 100% lethality was observed at P10, whereas in other backgrounds (e.g., 129/SvEv) ~50% of the CKO mice survived. However, the survivors displayed a fiber-type switching phenotype; i.e., myoblasts appeared normal, but differentiation from myoblasts to myotubes was blocked. 

The *MyoG-Cre:Smyd1* and *Mef2c* CKOs share a number of similarities. For example, we observed an abundance of primary myogenic cells but also the appearance of large multinucleated (fused) myoblasts that had failed to elongate (Figure 2(B,Cf,j)). This suggested that CKO myoblasts did not properly differentiate into myotubes—an event controlled to a great extent by MYOD1 and MYOG [22]. However recent reports also have linked similar highly polarized fusion deficiencies to melanoma cell adhesion molecule (MCAM) [22,25,26]. MCAM asymmetrically distributes at the tip of elongating myotubes where it colocalizes with actin binding and cell polarity regulators to facilitate directional elongation.

A possible disruption unrelated to MRFs is SMYD1 enzymatic function. SMYD1 is a muscle- specific histone methyltransferase [5,27] that is capable of globally altering the transcriptome post-transcriptionally, while yielding no apparent alteration in transcript abundances. This is further evidenced by the lack of gene expression differences in *Smyd1* WT and CKOs as determined by microarrays of both E12.5 and E14.5 muscle (T.L.R. and H.O.T; data not shown). Perhaps SMYD1 functions here by posttranslational methylation—an event that has been documented in a number of studies [5,27]. However, there is no evidence that any of the MRFs are methylated nor otherwise posttranslationally modified in a manner which would be catalyzed directly by SMYD1. 

### 3.6. Central Regulators of BAT are Unaffected by SMYD1 CKO 

A striking phenotypic feature of the *MyoG-Cre:Smyd1* CKO was loss of regional BAT (Figure 2B and Figure 3). Transcriptional control of BAT formation has been analyzed extensively [28]. The TFs peroxisome proliferator-activated receptor gamma (PPARγ) [29,30] and the CCAAT/enhancer-binding proteins (C/EBPα/β/δ) [31] are essential transcriptional regulatory components that precede the formation of BAT. A downstream consequence of these pathways is de novo mitochondrial biogenesis activation [28]. 

As shown in Figure 4, RT-qPCR analyses detected no alteration of any of these factors in *Smyd1-MyoG* CKO muscle relative to controls. Thus, classical mitochondrial BAT biology appears unscathed by loss of SMYD1.

### 3.7. SMYD1 Regulates Immune Factors Critical for BAT Development and Physiology

Alternatively, regulation of BAT metabolism by both the innate and the adaptive arms of the immune system has been well established [32,33,34,35]. Recently, the impact and the molecular characterization of BAT-associated T regulatory (Treg) cells have been defined [36]. Several pro-inflammatory markers are induced by Treg-deficient BAT, including the cytokine interleukin 6 (IL-6), the chemokine ligand 2 (CCL2), and tumor necrosis factor alpha (TNFα) [37]. 

As anticipated from those reports, several previously demonstrated Treg-BAT factors were upregulated by SMYD1 loss (Figure 4). These include IL-6, CCL2, TNFα, and several additional pro-inflammatory chemokines (CCL6, 7, and 9). Also, as shown previously [36], we observed modest upregulation of *Foxp3*, the central transcriptional regulator of Treg differentiation, and several of its direct target genes, including BCL11, RUNX1, and CBFβ. Both BCL11 and RUNX1 also have been shown to reciprocally regulate *Foxp3* [38]. 

The relevance of TNFα and IL-6 modulation with respect to muscle biology is particularly striking. TNFα is essential for incorporating fatty acids into muscle tissue, whereas there is a direct correlation between IL6 and fatty acid oxidation in skeletal muscle [39]. Both are inflammatory cytokines required for the maintenance and differentiation of skeletal muscle. TNFα plays a bipolar role in muscle regulation. In its absence, myogenic differentiation is blocked by failure to activate p38 [40]. In contrast the absence of TNFα strongly contributes to cachexia, or wasting atrophy [41]. Overexpression and knockdown experiments have documented that the myogenic markers Myog, α-actin, and p21 directly correlate with IL-6 levels [42]. Although TNFα levels are extremely low in SMYD1 CKO muscle (Figure 4), they are not strictly dependent on SMYD1 KD levels (Figure S4). We also observed a tight negative correlation of IL-6 levels with gender at E13.5 (Figure S4). 

### 3.8. SMYD1 as a Potential Determinant of Muscle vs. Adipose Fate 

Skeletal muscles (slow and fast twitch) and fat tissues (white, beige, and brown) differ broadly in morphology, anatomical locations, transcriptional profiles, and physiologic function [43,44]. BAT and skeletal muscle derive from the same multi-potent progenitors that originate within the central dermomyotome [45,46]. Expression of PAX7, an essential multi-potent progenitor TF, is turned off in dermal and BAT but continues to be expressed in MYOD+ embryonic and postnatal muscle progenitors [47,48].

It was shown recently that two MRFs, MyoD and Myf5, act downstream of PAX7 to repress the BAT determining factor, PRDM*16*, thereby acting as a “molecular switch” to repress BAT fate [48]. 

To determine if SMYD1 might be a component of this fate-switch circuit, we compared the levels of the above three components in E12.5 WT and CKO embryos. While we observed no effect on PAX7, MYOD nor MYF5, CKO skeletal muscle was depleted of PRDM16—a quintessential requirement for BAT fate choice (Figure 4). 

These data suggest that SMYD1 may control muscle vs. adipose fate via modulation of *Prdm16* via a pathway different from that described for PAX7. Loss of PRDM16 in adipose tissue results in significant upregulation of thermogenic genes, including the brown fat-uncoupling protein, UCP-1 [49], which previously was touted as a “master regulator” of conventional BAT [28,29,30], and cell death activator-A (CIDE-A), which controls metabolic rates and higher lipolysis in BAT [50]. Yet as shown in Figure 4, both of these downstream modulators of PRMD16 are downregulated in *MyoG-Cre/Smyd1* muscle. This result was the opposite to what would be expected if SMYD1 directly repressed PRDM16, as levels of these direct PRDM16 targets would be upregulated. An alternative mechanism is therefore required.

One possibility is that SMYD1 independently regulates transcription of PRDM16, UCP-1, and CIDE-A. Or, perhaps SMYD1 executes *Prdm16* activation via transcriptional or posttranscriptional control. Finally, SMYD1 may mediate cross-talk with adipogenic energy stores or absorption of nutrients. Alternatively, if Smyd1-deficient multipotent progenitors are incapable of signaling the break-down of BAT to release metabolites, BAT would accumulate more efficiently in CKOs than in control embryos.

None of these hypotheses explain why the proliferation marker, Ki67, stained BAT more densely in SMYD1-deficient muscle (Figure 3). One explanation is that while PAX7 is repressed in normal BAT, it continues to be expressed in proliferating muscle progenitor cells in embryonic and young postnatal muscle that give rise to BAT [51,52]. Enlarged BAT foci also have been attributed to increased proliferation and differentiation of brown pre-adipocytes [53]. Alternatively, the observation might have a physiologic explanation. For example, SMYD1-deficient muscle might be unable to efficiently absorb nutrients. In this scenario, excess nutrients within the system could be adsorbed by brown adipose, resulting in abnormal proliferation and expansion of BAT.

### 3.9. SMYD1 Controls Specification of Muscle Fiber-Type in Rare Adult Survivors 

**Figure 5:**
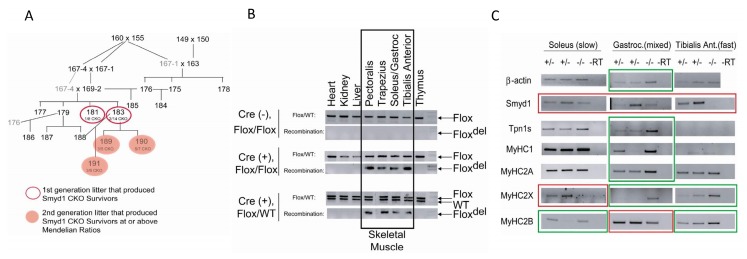
SMYD1 controls specification of muscle fiber-type in rare adult survivors. (**A**) A heritable change within the *Smyd1* locus allowed some *MyoG-Cre/Smyd1* (CKO) mice to survive. Mice were bred in a non-gender specific manner by intercrossing *Smyd1; Cre^Flox/Flox^* mice. Two of four litters from a mother-son cross yielded several CKO pups of this genotype. Further crossing of these lines produced CKO pups at or above expected Mendelian ratios. Pups within a litter are indicated in open (first generation) and orange (second generation) circles. Numbers in gray are duplications necessary to show breeding within multiple partners. (**B**) Recombination occurs specifically within skeletal muscle of *MyoG-Cre*+ *Smyd1^Flox/Flox^* (CKO) survivors. Mice were sacrificed, DNA was extracted from their tissues and analyzed via endpoint PCR at postnatal day (P12). Recombination specifically is confirmed as both the *Cre* transgene and *Smyd1^flox^* allele are present. (**C**) Slow/fast fiber type composition is altered in adult *Smyd1*-deficient skeletal muscle survivors. Mice were sacrificed, RNA was prepared and subjected to end-point RT-PCR analysis. Selective isolation of slow vs. fast muscle is indicated by absence of the slow markers, Troponin1 and MyHC1, in the fast muscles. Heterozygotes express variable levels of SMYD1 (e.g., compare values of all left-side columns to those in the middle). *Smyd1* KO in the soleus muscle is less efficient than in other muscles. Expression levels of MyHC2X and MyHC2B are altered in all three muscle types. In slow muscle, MyHC2x expression levels directly correlate to those of SMYD1, whereas in mixed (slow + fast) gastrocnemius, MyHC2x is indirectly correlated with SMYD1 levels. MyHC2B levels are indirectly correlated with SMYD1 levels in slow and fast tissues, whereas they are directly correlated in mixed tissue. These results are representative of three independent measurements.

After mating and genotyping over 190 litters of *MyoG-Cre/Smyd1* mice, we observed no postnatal CKOs on the non-isogenic background described in the Materials and Methods section. However, two of four litters produced by one particular mother-son cross yielded pups in which CKOs survived and appeared outwardly normal into adulthood—albeit not at normal Mendelian ratios (Figure 5A). 

DNA analyses revealed that a muscle-specific recombination event had occurred within the *Smyd1* locus. The mutation(s) did affect SMYD1 expression at the RNA level, perhaps because *MyoG-Cre* expression was lower than in age-matched control embryos (Figure 5B). Because our mouse lines were not isogenic, this particular branch may over time have lost or gained a genetic or epigenetic modification(s) that delayed/reduced *Cre* expression. If so, the data indicate that SMYD1 is not required for skeletal muscle function per se but is essential for maintaining the development and differentiation of skeletal muscle. This is supported by the *Myf6-Cre* data reported previously [12,13] and noted below.

Previously unpublished data obtained from shRNA knockdowns of SMYD1 in C2C12 myocytes indicated that SMYD1 repressed Calcineurin (CaN) regulation of slow fiber formation (L. Zhu, personal communication). Consistent with this was our previous observation that SMYD1 forms a stable complex with CaN [54]. We further showed that knockdown of SMYD1 led to dissociation of the complex and activation of CaN-mediated dephosphorylation [54]. However, and in apparent conflict with these conclusions, ablation of *Smyd1* postnatally at 6 wk via *Myf6-Cre* produced mutant mice that exhibited loss of fast-twitch muscle markers [13]. 

The opportunity afforded by the rare adult *MyoG-Cre/Smyd1* skeletal muscle survivors allowed us to address this apparent paradox by providing adequate amounts of dissectible, regional-specific muscle groups of known fiber types. These include the soleus muscle, which is composed primarily of slow muscle fibers; the tibialis anterior, primarily fast muscle fibers; and the gastrocnemius, which is a mixture of the two fiber types [55,56]. In skeletal muscle, there are four types of myosin heavy chains: a slow oxidative *MyHC1*, a fast oxidative *MyHC2A*, a fast/intermediate *MyHC2X*, and a fast glycolytic *MYHC2B* [55,56]. 

As shown in Figure 5C, at postnatal day 12 (P12), SMYD1 knockdown was robust within gastrocnemius and within the tibialis anterior muscles, but less effective in the soleus (probably because *Cre*-expression there was less efficient (Figure 5C). Surprisingly, based on previous observations, we detected no expression of slow muscle markers (MyHC-1 and TPN1S) within CKO fast tibialis anterior muscles, nor in these markers (including MyHCIIA) in either in the tibialis anterior or the soleus. However, glycolytic fast muscle myosin (MyHC-IIB) was upregulated in CKO slow soleus (Figure 5C). Alternatively, intermediate oxidative-glycolytic myosin (MyHC-IIX) was decreased in the slow soleus but increased in the mixed gastrocnemius and fast tibialis anterior fibers of *Myog-Cre:Smyd1* CKOs (Figure 5C). These results were confirmed in SMYD1-deficient soleus at P24 both at the RNA level (Figure S5) and at the protein level for a pan antibody against MyHC-IIX that recognizes all slow twitch fibers (Figure S6).

We suspect these phenomena were due to epigenetic silencing, as such, phenomena typically are not “all or none”. This is evidenced by the mechanism of reversible methylation, which plays a role in many adaptive processes [57]. In the present context, such a process could lead to incremental decreases in *Cre* transgene expression from generation to generation. One might expect such alterations to be both stochastic (i.e., a random percentage acquires a change) and inheritable (i.e., once acquired, reversion is unlikely in subsequent generations).

These data, as with our previous reports [12,13], suggest that loss of SMYD1 results in slow-to-fast transformation of muscle fiber-type in the soleus. Physiologically, loss of slow-twitch, mitochondrial-rich fibers would lead to reduction in oxidative metabolism and greater fatigue. If mitochondrial-rich slow fibers are compromised, *Myog-Cre:Smyd1* CKO mice would rely on type II^a^ fast fibers to balance this deficiency. Accordingly, MyHC^2a^ and MyHC^2b^ muscle function should remain unaffected or perhaps even slightly increased on loss of SMYD1. Muscle fiber composition is a critical determinant of the capacity for mitochondrial fuel oxidation, ATP synthesis, and muscle endurance [56,58]. Thus, it would be informative to subject *Myog-Cre:Smyd1* adult survivors to treadmill and distance running tests.

## 4. Concluding Remarks

We find it remarkable that SMYD1 deficiency leads to a number of multiple phenotypes dependent on the developmental stage and/or the Cre-recombinase employed for targeting [5,6,7,8,9,10,11,12,13]. Is there a single fundamental mechanism underlying this apparent heterogeneity? We submit that, yes, the diverse consequences of SMYD1 loss depends upon its bi-functionality as both a lysine (K) and a histone (H) MTase. Further diversification most surely rests on its ability to catalyze multiple histone marks, including H3K4m3, H3K4m2, and H4K20m2, as well as a growing list (currently up to nine) of confirmed KMTas targets [4,5]. While no additional “direct” targets were identified in this report, we judge it very likely that much, if not all, of the transcriptional changes documented in Figure 4 result from protein stabilization or other post-transcription functions. We suggest that identification of additional methylation targets and systematically assigning them into specific developmental stages of heart and skeletal muscle development will be key in establishing the basis for SMYD1-deficient heterogeneity. 

While we feel that our study opened another new avenue for SMYD1 research, additional work is required to better interpret the complex phenotype. For example, a systematic histologic time course across each stage of prenatal development needs to be linked to that for full gene expression analyses. At present, results are compromised as only certain stages were analyzed, and there may be some discrepancy between the histology (most penetrant at E14.5) and the gene expression (analyzed primarily at E12.5), since the authors suggest an increase in BAT instead of muscle (at E14.5), but the gene expression shows a decrease in expression of BAT markers, Prdm16 and UCP1, at E12.5. There are also lots of data mentioned but not included. A full time course analysis will make it possible to fully interpret what appears to be an interesting and unusual phenotype.

## Figures and Tables

**Table 1:** Skeletal muscle-specific SMYD1 CKO does not alter Mendelian ratios prior to birth.

Age (DPC)	*Smyd1* MT	*Smyd1* HET	*Smyd1* WT	Total
9.5	5	11	6	22
12.5	4	9	10	23
13.5	2	3	3	8
14.5	0	4	0	4
15.5	6	11	8	25
16.5	6	5	2	13
17.5	2	4	3	9
Total	25	47	32	104
Expected Ratio	0.25	0.5	0.25	
Observed Ratio	0.24	0.45	0.31	

WT (*Smyd1* WT/Flox/MyoG-Cre) were crossed with MT (*Smyd1* WT/KO/MyoG-Cre); HET (both *Smyd1* KO/WT and *Smyd1* Flox/WTCre). The ratio of MT: HET: WT is summed and shown for all stages.

## Supplementary Materials

The following are available online at http://www.mdpi.com/2079-9721/7/1/1/s1, Figure S1: RT-PCR primer pairs and conditions. Figure S2: Primer sequences used for qPCR. Figure S3: Skeletal muscle *Smyd1* deficiency does not affect transcript levels of Myogenic Regulatory Factors (MRFs). Figure S4: Transcript levels of TNFα and IL6 show gender variation in Smyd1 deficient skeletal muscle. Figure S5: Slow and fast muscle markers are altered. Figure S6: slow muscle fibers are reduced.
