# Supplementary material for: Loss of SMYD1 Results in Perinatal Lethality via Selective Defects within Myotonic Muscle Descendants
Source: Diseases. 2018 Dec 20;7(1):1. doi: 10.3390/diseases7010001 (PMC6473627; doi:10.3390/diseases7010001)
Supplement: Supplementary file 1 [file diseases-07-00001-s001.pdf]

SUPPLEMENTAL FIGURES

Loss of SMYD1 results in perinatal lethality via selective defects within myotonic muscle descendants.

S-Figure 1. RT-PCR primer pairs and conditions

Gene	Forward Primer	Reverse Primer	Annealing Temp.	Cycle# (range)	Produ[illegible] Size(b[illegible]
1700129I15Rik	ggccctgaagctaactgaagac	ggtttccttggagggcagag	65	36-41	169
Actb	tacgagggctatgctctc	cgcagctcagtaacagtc	58-62	19-30	651
Akt1	gccctggactacttgcac	cgaacagcttctcgtggtcc	61	27-43	300
AR	caagctggagaacccattggac	ggcagcaaaggaatctggttgg	55	35	897
Calm4	gcagcagactgacatccagttg	cacacccaaggaaaccatcctc	65	30-35	745
CideA	gcctgcaggaacttatcagc	cacggccttgaagcttgtg	58	30	347
Cre	ggacatgttcagggatcgccaggcg	gcataaccagtgaaacagcattgctg	60-62	20-35	268
Cyr61	ggcatctccacacgagttacc	ccgcatcttcacagttctggtc	60-65	30-40	268
Gyltl1B	cgtcgccagtgctctctatg	cagctgaccactgcacactg	60	30-32	414
Hand2	tacttccacggctggcttattg	tcgttgctgctcactgtgcttt	62	35-36	467
Igf1	cttctacctggcgctctgc	gtcttgggcatgtcagtgtgg	61-61.4	27-43	300
IL6	gccttcttgggactgatgc	cgcactaggtttgccgag	60	27-35	604
Irx4	tgctggccatcatcaccaagat	ttggaccttggcgtttcagcat	62	35-36	666
Irx5-2	caccaagatgaccctcaccc	gtaataaagaggccgggacagc	60-62	34-36	763
Isl1	tttctccggatttggagtggca	atgcaagggactgagagggtct	62	35-40	359
Kcnd2	tttcgtgcgcagtgtcatgagt	aagttcgacacgatcacaggca	62	28-35	459
Mafbx	gtctgtgctggtgggcaac	gcaaagctgcagggtgac	61	30-43	394
Mdfi	cggaagttgcagacgcatcc	gagtccacagcactccatg	60-65	30-40	342
Mef2c	acagcaccaacaagctgttcca	tgcttaaagagcaaccggagga	62	30-35	549
MK2	gtgcctgctgattgtcatgg	gatgacacccaaggaccac	60	30-32	361
Murf1	ctcctgcagagtgaccaag	gtcccaaagtcaatggccc	61	30-43	399
Myf5	ttcggagcacacaaagctga	aagctgctgttctttcggga	60	30-35	431
Myf6	tgcgcgaaaggaggagac	gaaaggcgctgaagactgc	60	32-35	379
MyHCIIa (Myh2)	cgaccctgaccgtgaaag	tccggtgatcaggattga	60	38-40	335
Myo6	gtggagcaggcaaacaatgctc	cttcattaacacccaggccctc	60-65	36-40	280
MyoD1	tgatgacccgtgtttcgact	ttccctgttctgtgtcgctt	60	32-37	831
MyoG	aagcgcaggctcaagaaagt	tcagttgggcatggtttcgt	60	30-35	426
Mb	ctcaccctgagaccctgg	gtcattccggaagagctcc	60-65	30-37	330
Myostatin	gctggcccagtggatctaaatg	gggtgtgtctgtcaccttgac	60	30-32	362
p21	gcagatccacagcgatatccag	ctaagggccctaccgtcctac	60	28-30	768
p38	gagaacgtcattgggcttctgg	gtgttgtttccattcgtccacgc	60	30-32	780
PW1	catgagccgaagtgggagag	gtggccttgtgtcatgtgag	60	25-32	399
Serca2	ggcatctttgggcaggatg	cctcaacagcagccacaatg	60	30-32	1239
Serpini1	gtgatggatccaatgaggctgg	gcacagccatcctactaatcgc	60-65	40-41	411
skNAC	ccagccgatcctcgtcaac	ccttccttgccttcttctcac	60-65	29-37	260
Smyd1(1-6)	atggagaacgtggaggtcttc	gctcacaggagcagtcaaagtag	60-62	25-29	816/42[illegible]
Smyd1(2-6)	ctgcgggcagtgcaagtt	gctcacaggagcagtcaaagtag	60-62	25-37	640
Smyd1(4-6)	caactgcaacggtttcactctcag	gctcacaggagcagtcaaagtag	60-62	30	260/22[illegible]
Smyd1(6-9)	acatagacttcctgcacctcagtg	catgggtcaccaggagaatagcat	60-62	30	504
Smyd1(7-9)	cgagggtttgtatcacgaggttgt	catgggtcaccaggagaatagcat	60-62	30	300
Smyd2	ctggacaagctagacaacg	tgatcttctgcccgtatttcaggg	62	28-32	700
Smyd3	gagtcgtggcgtagtctgtgatc	cagctgagacgcatcctggatc	62	28-32	360
Sprrl3	cactcaccttccgaggtatcc	ctgccaccactgctacaac	65	36-41	365
Sprrl3	cactcaccttccgaggtatcc	ctgccaccactgctacaac	65	36-41	365
TNFa	ggcctccctctcatcagttc	gagatagcaaatcggctgacgg	60	25-35	293
Tnni1	ggagcaggaacacgagga	actggccgctccttttct	60	35-37	377
UCP-1	gaaggtcagaatgcaagccc	catggacatcgcacagcttg	58	30	364
Zfp36	ccatggatctctctgccatctacg	caggcctggttagggtctc	60	25-35	724
Zfy	tcctgagcatgagcaacagatgga	ctgactggtactgtttggattcagg	60	31	217

Figure S1. RT-PCR primer pairs and amplification conditions. PCR primers for all RT-PCR were designed and optimized using online Primer3 software (http://frodo.wi.mit.edu/cgi-bin/primer3/primer3_www.cgi). Details of conditions are provided in Methods and Materials.

S-Figure 2

S-Figure 1. RT-PCR primer pairs

Muscle Regulatory Factors

MyoD: (F) 5′-TACAGTGGCGACTCAGATGC-3′ and (R) 5′-GAGATGCGCTCCACTATGCT-3′
MyoG: (F) 5′-CTACAGGCCTTGCTCAGCTC-3′ and (R) 5′-ACGATGGACGTAAGGGAGTG-3′
Mef2C: (F) 5′-GCCGGACAAACTCAGACATT-3′ and (R) 5′-TGGGATGGTAACTGGCATCT-3′
Myh7: (F) 5′-CTACAGGCCTGGGCTTACCT-3′ and (R) 5′-TCTCCTTCTCAGACTTCCGC-3′
Myh2: (F) 5′-ATCCAAGTTCCGCAAGATCC-3′ and (R) 5′-TTCGGTCATTCCACAGCATC-3′
Myh1: (F) 5′-ATGAACAGAAGCGCAACGTG-3′ and (R) 5′-AGGCCTTGACCTTTGATTGC-3′
Myh4: (F) 5′-AGACAGAGAGGAGCAGGAGAGTG-3′ and (R) 5′-CTGGTGTTCTGGGTGTGGAG-3′
Myh7: (F) 5′-AATTCCTTACTTGCTACCCTC-3′ and (R) 5′-CTTCTCAGACTTCCGCAG-3′
Myh7b: (F) 5′-AGAGTGTGGAGCAGGTGGTATT-3′ and (R) 5′-GGTCTGATTGATTCGAGAAACC-3′
Myh3: (F) 5′-TGAACAGATTGCCGAGAACG-3′ and (R) 5′-GGAGAATCTTGGCTTCTTCGTG-3′
Myh8: (F) 5′-ATCGTGAGAACCAGTCCATCC-3′ and (R) 5′-TTTGCCAGACTCCTCCTTCTTC-3′
Myf6 (F) 5′-GGAGCGCCATCAGCTACATC-3′ and (R) 5′ CGAGGAAGTCCGAGCCATT-3′
Myf5 (F) 5′-AGGAGGAGGCTGAAGAAAGTGA-3′ and (R) 5′-GCTCTGTCTCGGCAGGTGATA-3′
Pax3 (F) 5′-GGAGCCTGTGGACTTGGATCTATTAG and (R) 5′-GGTTGAGAAGTACCACGGGAAGAG-3′

Brown Adipose Tissue (BAT)

E2f4: (F) 5′-GATGCCTACTTAAAGAGAAACAGG and (R) 5′-CCACTATATTGCCCAAGAACC-3′
Pparγ (F) 5′-GACTCAGGGACAGAGTGAGG and (R) 5′-CGGTAGTTCTGGAGACCTGG-3′
Pparα (F) 5′-GCGTACGGCAATGGCTTTAT and (R) 5′-GAACGGCTTCCTCAGGTTCTT-3′
Rbl1 (F) 5′-CGAACTGACAGTGGGAGTCTTC and (R) 5′-TCTCTTAGCACTCCCTGCGGTA-3′
Klf11 (F) 5′- ATGGATGCAGCCACACCTGAAC and (R) 5′-GGAGAAACAGGTGTCCTTGTCG-3′
C/Ebpα (F) 5′-TGGACAAGAACAGCAACGAGTAC and (R) 5′-GCAGTTGCCCATGGCCTTGAC-3′
C/Ebpβ (F) 5′-GGTTTCGGGACTTGATGCA and (R) 5′-CAACAACCCCGCAGGAAC3′
C/Ebpγ (F) 5′-CCCCAAAGCTATGTGCCTTTC and (R) 5′-CCTGGAGGGTTTGTGTTTTCTG -3′

Immune BAT

TNF-α (F) 5′-ATGAGCACAGAAAGCATGA and (R) 5′-AGTAGACAGAAGAGCGTGGT-3′
IL-6 (F) 5′-CCTCTGGTCTTCTGGAGTACCi and (R) 5′-ACTCCTTCTGTGACTCCAGC-3′
Ccl6 (F) 5′-GTGGCATAAGAGAAGCAGCAG and (R) 5′-TCTATCCTTGTGGCTGTCCTTGG-3′
Ccl7 (F) 5′-AAGATCCCCAAGAGGAATCTCAAG and (R) 5′- CAGACTTCCATGCCCTTCTTTG-3′
Ccl9 (F) 5′-GATGAAGCCCTTTCATACTGC and (R) 5′-GTGGTTGTGAGTTTTGCTCCAATC -3′
Rank (F) 5′-CTGCTCCTCTTCATCTCTGTG and (R) 5′-CTTCTGGAACCATCTTCTCCTC-3′
Foxp3 (F) 5′-CTGAGGTTTGGAGCAGAAGGA and (R) 5′-TCTGAAGCCTGCCATGTGAA-3′
Bcl11A (F) 5′-GTCTGCACACGGAGCTCTAA and (R) 5′-CACTGGTGAATGGCTGTTTG-3′
Runx1 (F) 5′-CTCGGCAGAACTGAGAAATG and (R) 5′-GGTGATGGTCAGAGTGA-3′
Cbfβ (F) 5′-TGAAGAGGCTCGGAGAAGGACA and (R) 5′-CGAAGTTTGAGGTCATCACCACC-3′

Muscle vs adipose fate

Pax7 (F) 5′-ATCAGTGGGTGCCTTACCAA and (R) 5′-GCCAAATGTTTACTGGGACA-3′,
Prdm16 (F) 5′-CGACGAAGAGGATGATGAACAC and (R) 5′-TCCCTAGCATTGTCAGTTTGGA-3′,
Ucp-1 (F) 5′-CGACTCAGTCCAAGACTACTTCT and (R) 5′-GCGGCTGAGTATCTTGTTTC-3′,
Cide-A (F) 5′-GGGCAGTGATTTAAGAGACGCTT and (R) 5′-CCACAGCCTATAACAGAGAGCAG-3′,

Figure S2. RT-qPCR primer pairs. PCR primers for real-time, quantitative (q) PCR were designed using the online Primer3 software (http://frodo.wi.mit.edu/cgi-bin/primer3/primer3_www.cgi). Details of conditions are provided in Methods and Materials.

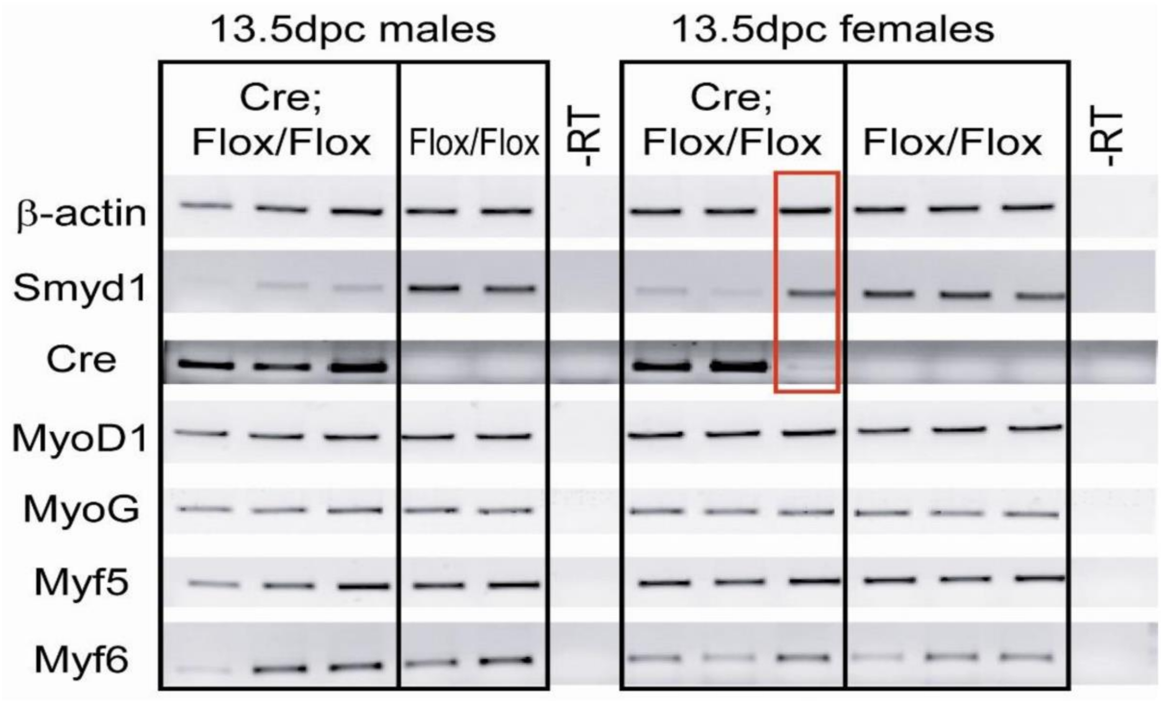

Figure S3. Skeletal muscle SMYD1 deficiency does not affect transcript levels of Myogenic Regulatory Factors (MRFs) at E13.5. Limbs obtained from E13.5 embryos were homogenized in Trizol and genotyped for *Cre*, *Smyd1*, and gender. Appropriate samples were reverse transcribed using primers listed in S-Fig. 1. Boxed samples are genotypically *Cre*+, but did not show significant reduction because SMYD1 levels were not strongly reduced. These data were reproduced in Figure 4 using RT-qPCR.

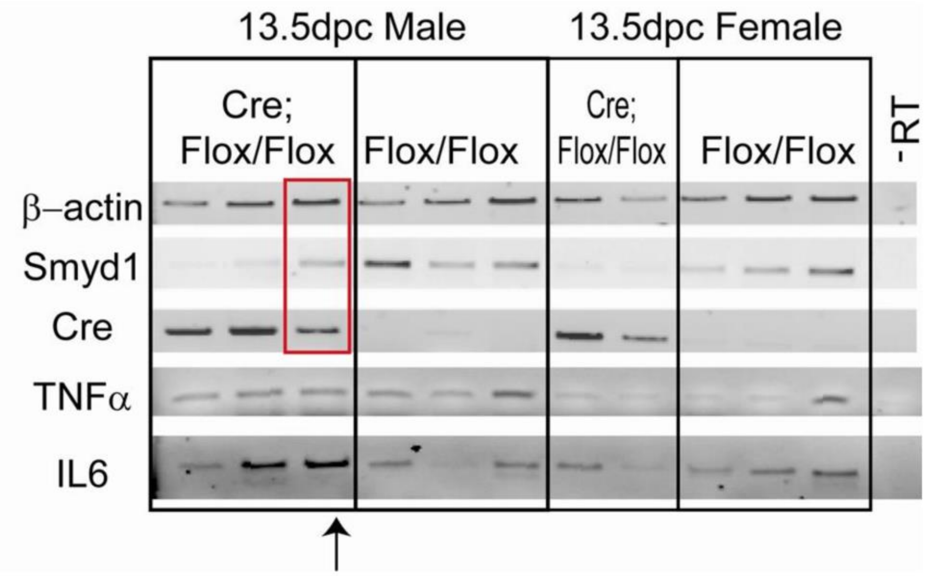

Figure S4. Transcript levels of TNFα and IL6 show gender variation in SMYD1-deficient skeletal muscle. Limbs obtained from E13.5 embryos were homogenized in Trizol reagent and genotyped for *Cre, Smyd1*, and gender. Appropriate samples were subjected to endpoint RT-PCR. Some genotypically *Cre*+ (boxed) do not express well leading to modest reduction of SMYD1 levels (boxed). TNFα transcripts vary, but are more dependent on gender than on SMYD1 levels, whereas IL-6 transcript variance correlates with SMYD1 levels. SMYD1 deficiency in males results in increased levels of IL-6, whereas SMYD1 deficiency in females leads to slightly decreased levels of IL6.

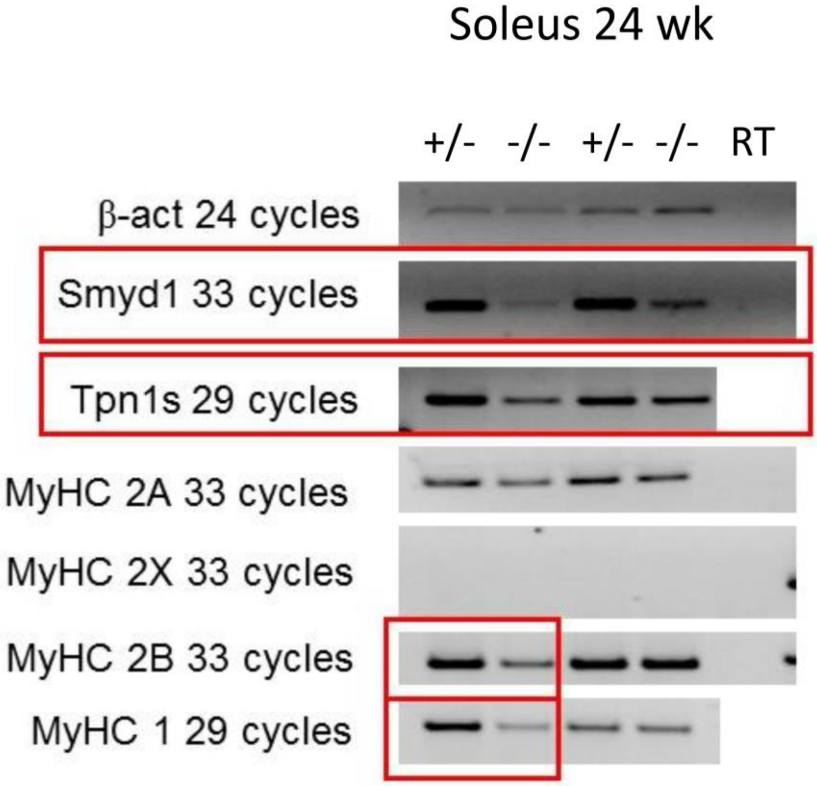

Figure S5. Both slow and fast twitch markers are downregulated in SMYD1-deficient soleus muscles at P24. RT-PCR performed on limbs from 2 different CKOs (-/-) and WT litter mates (+/-) reveal reduction of slow twitch (Tpn1s and MyHC1) as well as fast twitch (MyHC2B) markers following loss of SMYD1.

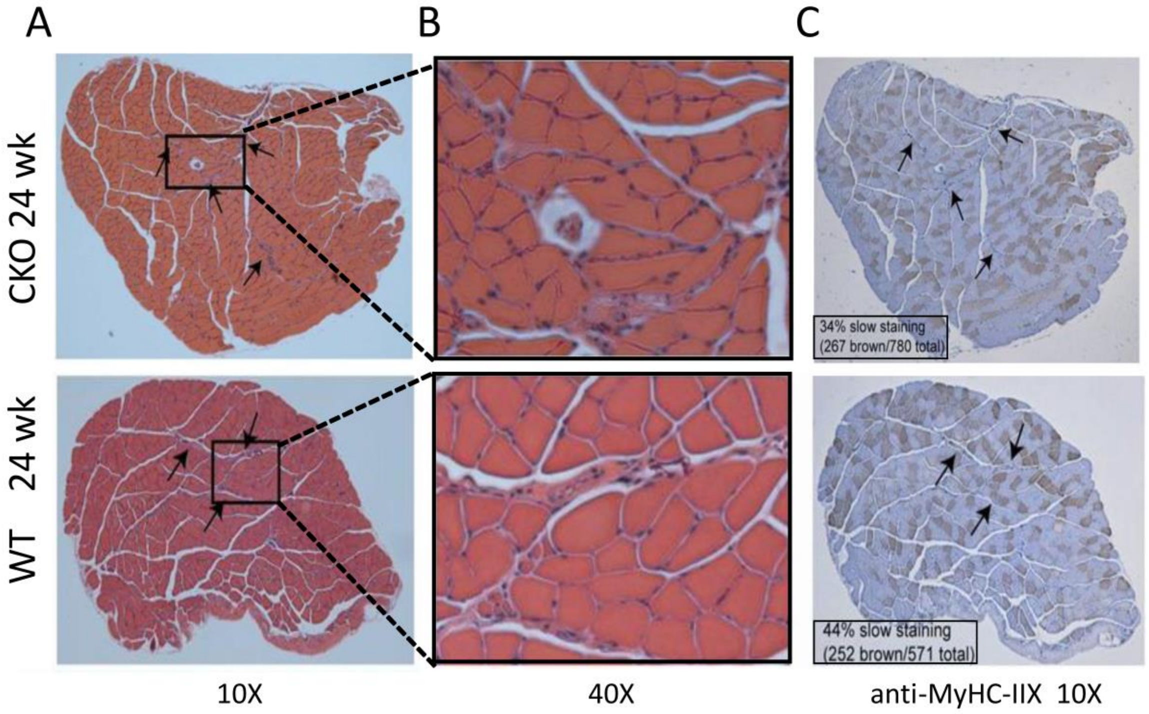

Figure S6. Confirmation of reduced slow twitch protein in 24 day SMYD1-deficient soleus. (A) Transverse sections at 10X magnification of P24 week soleus. Arrows denote capillaries and influx of inflammatory cells (blue) which are more apparent in the CKO. **(B)** Blow up (40X) of the region boxed in (A). **(C)** Immunostaining of transverse section of (A) with a pan-anti-slow twitch Ab against MyHC-

IIX (mAb GTX11083; GeneTex, Inc) indicates reduction of overall slow twitch fiber density of ~10% as calculated by the number of MyHC-IIX-positive cells divided by total number of cells (boxed). Arrows indicate influx of inflammatory cells (blue).
